# MicroRNA profiling of diverse endothelial cell types

**DOI:** 10.1186/1755-8794-4-78

**Published:** 2011-11-02

**Authors:** Matthew N McCall, Oliver A Kent, Jianshi Yu, Karen Fox-Talbot, Ari L Zaiman, Marc K Halushka

**Affiliations:** 1 Department of Biostatistics and Computational Biology, University of Rochester, Rochester, NY, USA; 2 Department of Pathology, Division of Pulmonary and Critical Care Medicine, Johns Hopkins University SOM, Baltimore, MD, USA; 3 Department of Medicine, Division of Pulmonary and Critical Care Medicine, Johns Hopkins University SOM, Baltimore, MD, USA

**Keywords:** miR-99b, miR-20b, let-7b

## Abstract

**Background:**

MicroRNAs are ~22-nt long regulatory RNAs that serve as critical modulators of post-transcriptional gene regulation. The diversity of miRNAs in endothelial cells (ECs) and the relationship of this diversity to epithelial and hematologic cells is unknown. We investigated the baseline miRNA signature of human ECs cultured from the aorta (HAEC), coronary artery (HCEC), umbilical vein (HUVEC), pulmonary artery (HPAEC), pulmonary microvasculature (HPMVEC), dermal microvasculature (HDMVEC), and brain microvasculature (HBMVEC) to understand the diversity of miRNA expression in ECs.

**Results:**

We identified 166 expressed miRNAs, of which 3 miRNAs (miR-99b, miR-20b and let-7b) differed significantly between EC types and predicted EC clustering. We confirmed the significance of these miRNAs by RT-PCR analysis and in a second data set by Sylamer analysis. We found wide diversity of miRNAs between endothelial, epithelial and hematologic cells with 99 miRNAs shared across cell types and 31 miRNAs unique to ECs. We show polycistronic miRNA chromosomal clusters have common expression levels within a given cell type.

**Conclusions:**

EC miRNA expression levels are generally consistent across EC types. Three microRNAs were variable within the dataset indicating potential regulatory changes that could impact on EC phenotypic differences. MiRNA expression in endothelial, epithelial and hematologic cells differentiate these cell types. This data establishes a valuable resource characterizing the diverse miRNA signature of ECs.

## Background

MicroRNAs (miRNAs) are highly conserved ~22 nt long regulatory RNAs. Critical modulators of post-transcriptional gene regulation, miRNAs bind to 3' UTR regions of mRNAs, where they function to block translation and decrease mRNA stability. To date, over 800 miRNAs have been identified in the mammalian genome. The diversity of these miRNAs and the regulatory roles they have in different cell types are just beginning to be explored.

Hundreds of studies have been performed investigating miRNA expression differences by array, deep RNA sequencing or qRT-PCR methods. Generally these studies either compare normal tissue to a diseased/malignant/perturbed state or developmental tissues over a time course [1-3]. There are fewer studies investigating miRNA expression patterns in normal tissues. A pioneering experiment investigated miRNAs from the heart, liver, spleen, small intestine, colon and brain of mice, identifying several "organ specific" miRNAs [4]. A second study of 24 human organs confirmed and expanded on these initial findings. It also demonstrated that miRNA expression was highly correlated to other miRNAs located within 50-kb of each other suggesting coordinated polycistronic miRNA expression in tissues [5]. While these and other studies have demonstrated miRNA organ specificity, they did not compare individual cell types. Most organs are comprised of a variety of cell types. For example, the small bowel is comprised of multiple types of epithelial cells, endothelial cells, smooth muscle cells, and inflammatory cells. Thus additional experiments are needed to tease apart the miRNA contributions of these different cell types. Endothelial cells (ECs), in particular, are located in all organs thus their miRNA expression patterns are not accounted for in whole tissue experiments. Exploring relative cell-specific miRNA patterns can help us identify variable regulatory control of miRNAs in different cell types.

The importance of miRNAs to endothelial cell activity has been demonstrated. The knockdown of Dicer, a miRNA processing enzyme, unexpectedly resulted in a severe attenuation of angiogenesis [6]. This was an early pivotal experiment in EC miRNA biology. A number of miRNAs have since been described that are expressed at high levels in the endothelium and regulate key genes and activities. Several studies, with advancing numbers of miRNAs evaluated, have provided a starting point for EC miRNA discovery [7-10]. MicroRNAs including miR-126, miR-19a, and miR-21 modulate genes such as VCAM-1, cyclin D1, and eNOS [6,7,11]. In turn these interactions regulate critical pathways of angiogenesis, response to shear stress, cellular proliferation and NO production [12-14]. While miRNAs are important in endothelial cell (EC) function, the similarity/differences of their expression patterns across a variety of EC types has not been established.

An EC's vascular bed of origin strongly affects its phenotype, gene expression, and protein expression. For example, variable cell-cell junction activity, orientation to flow, fenestration size, vesicle formation, and microvilli count are some of the molecular differences that explain how macrovascular ECs from the aorta are known to behave differently than microvascular ECs taken from the liver sinusoids [15]. Recent work by Bhasin et al, identified unique patterns of gene expression (mRNA) in 5 unstimulated cell cultures of ECs taken from macrovascular, microvascular, and venous locations [16]. In this study, mRNA expression patterns could be used to cluster EC types, differentiating microvascular and macrovascular types based on shared gene expression. Patterns of protein expression are also influenced by EC origin. A proteomic comparison of bovine aortic ECs, lymphatic ECs and venous ECs by MALDI-TOF identified numerous variably expressed proteins [17], again demonstrating diverse expression patterns of ECs from different vascular beds. Differences in miRNAs across these EC types are unknown.

Because phenotypic, genetic, and protein differences exist between ECs taken from different vascular locations, we hypothesized that miRNAs would also vary between these same ECs. We believed these miRNA differences would inform us of classes of ECs that may share similar regulatory mechanisms. We also sought to compare global EC miRNA expression patterns with cells of different lineages. This would establish patterns of miRNA that were shared or unique to ECs.

## Results

### Endothelial cell miRNA diversity

**Figure 1:**
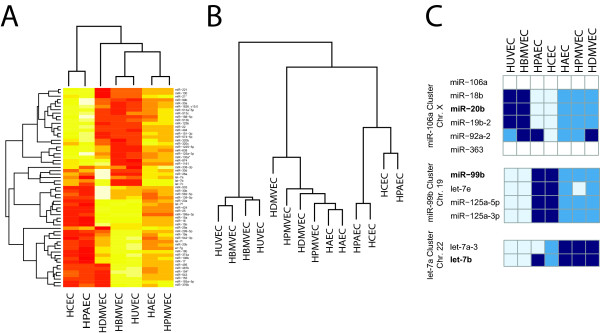
**Endothelial cell miRNA clustering.** (A) Hierarchical clustering based on LIMMA pairwise differential analysis of 59 miRNAs that had an unadjusted p value < 0.05. (B) Hierarchical cluster based on the SAM significant miRNAs let-7b, miR-20b and miR-99b. (C) Heat map demonstrating similar expression patterns across miRNAs within the same chromosomal cluster. Light blue represents low expression, dark blue represents high expression.

Total RNA was isolated from 7 primary EC cultures grown under identical conditions and hybridized to an Agilent V3 miRNA array. The array contained 843 human miRNAs, allowing us to determine a deep inventory of EC miRNA expression. After normalization, we identified 164 miRNAs expressed in ECs. Of these, 59 miRNAs (40%) were statistically variable between at least one comparison of EC types (ex. HUVEC vs HAEC) based on LIMMA pairwise differential expression analysis with an unadjusted p-value < 0.05 (Additional file 1, Table S1). Only 3 of these 59 miRNAs, let-7b, miR-20b and miR-99b, were also significantly different across all ECs as detected by the SAM algorithm, having 2.1, 1.6 and 1.8 fold variability respectively. We used the analyses (LIMMA significant and SAM significant) to generate both a hierarchical cluster heat map and an unsupervised Pearson Correlation based cluster map (Figure 1A, B). Both analyses yielded similar results. Human umbilical vein endothelial cell (HUVEC) and human brain microvascular endothelial cell (HBMVEC) cultures shared a similar miRNA signature and human coronary endothelial cells (HCECs) and human pulmonary artery endothelial cells (HPAECs) shared a similar miRNA signature. The human aortic endothelial cell (HAEC), human pulmonary microvascular endothelial cell (HPMVEC) and human dermal microvascular endothelial cell (HDMVEC) cultures were a third, less organized cluster.

### RT-PCR confirmation of EC miRNA array data

As validation of the SAM significant miRNAs, we performed RT-PCR for the mature miRNAs miR-99b, let-7b and miR-20b using TaqMan assays. RT-PCR Ct values were normalized to U6 snRNA for the mature miRNAs (Additional file 2, Figure S1). We compared the RT-PCR data to the pairwise comparison data in the miRNA array data set (Additional file 1, Table S1). We confirmed that of the 39 significantly different comparisons in the original data set, 27 (69%) were also significant (p ≤ 0.05) by unadjusted t-test in our RT-PCR data set (Additional file 1, Table S1).

### MicroRNA chromosomal cluster analysis

The three SAM significant miRNAs - miR-20b, miR-99b and let-7b - are located in miRNA clusters on chromosomes X, 19 and 22 respectively. We evaluated the relative expression patterns of the additional miRNAs in these clusters to determine if our data gave a signal of polycistronic regulation (Figure 1C). MiRNA-20b clusters with miR-18b, miR-92a-2, miR-19b, miR-363, and miR-106a. MiRNAs 18b, and 19b had expression patterns similar to miR-20b. MiRNA-92a-2 data is confounded by its homolog (miR-92a-1) on chromosome 13 whose signal cannot be differentiated by miRNA array [5]. We did not have data on miR-106a and miR-363. Let-7a, in a cluster with let-7b, shared a common expression pattern as did miRNAs let-7e and miR-125a (3p & 5p) both in a cluster with miR-99b (Figure 1C). This data suggested that entire miRNA chromosomal clusters are differentially regulated in ECs likely as polycistronic transcripts.

As a direct measure of the regulation of a polycistronic cluster, we attempted to investigate the expression of the primary transcripts (pri-miRNAs) for the miR-99b, let-7a (containing let-7b) and miR106a (containing miR-20b) clusters that encode these miRNAs. The RT-PCR primers for the miR-99b and let-7a primary transcripts were designed in exons found in an NCBI RNA reference sequence (Refseq) gene proximal to the miRNA clusters. No Refseq gene, human EST or mRNA proximal to miR-20b is known, preventing us from developing a suitable RT-PCR primer pair for detection of the miR-106a cluster. We observed a similar expression pattern of pri-miRNAs transcripts (normalized to β-actin) to the mature miRNAs let-7b and miR-99b demonstrating that these miRNA clusters are regulated from a common promoter region in these cell types (Additional file 2, Figure S1).

We then investigated the miRNA profile of all chromosomal clusters to determine if there were additional polycistronic transcripts that were variably expressed between EC lines with stronger signal than individual miRNAs. We found 27 cross-EC comparisons, from 9 miRNA chromosomal clusters, in which overall polycistronic expression was statistically different in pairwise comparisons between EC types. After removing individually significant miRNAs (based on LIMMA analysis - Additional file 1, Table S1) from these 27 comparisons, 22 comparisons, in 8 unique miRNA chromosome clusters remained significantly different (Table 1).

**Table 1:** Significant differences between EC types by miRNA cluster groups

Cluster	Comparison	Z. score	Adj. P. value
miR-512	HUVEC-HDMVEC	-5.49	5.7E-05
	HUVEC-HPAEC	-5.23	2.4E-04
	HUVEC-HCEC	-5.13	4.0E-04

miR-379	HDMVEC-HBMVEC	-7.76	1.2E-11
	HUVEC-HDMVEC	6.79	1.5E-08
	HCEC-HBMVEC	-6.56	7.3E-08
	HPAEC-HBMVEC	-5.99	3.0E-06
	HUVEC-HCEC	5.60	2.9E-05
	HPMVEC-HDMVEC	5.23	2.3E-04
	HUVEC-HPAEC	5.03	6.9E-04

miR-17	HUVEC-HPAEC	4.21	3.6E-02

miR-424	HPAEC-HDMVEC	-4.53	8.2E-03
	HUVEC-HPAEC	4.23	3.2E-02
	HDMVEC-HCEC	4.18	4.0E-02

miR-99b	HUVEC-HCEC	-5.42	8.2E-05
	HCEC-HBMVEC	5.07	5.6E-04
	HPAEC-HDMVEC	4.58	6.5E-03

miR-106b	HUVEC-HPAEC	4.19	3.9E-02

let-7a	HUVEC-HDMVEC	-5.11	4.4E-04
	HDMVEC-HBMVEC	4.99	8.5E-04
	HPAEC-HDMVEC	-4.61	5.7E-03

miR-15b	HPAEC-HDMVEC	-4.25	2.9E-02

**miR-512 cluster: **miR-512, miR-1323, miR-498, miR-520e, miR-515, miR-519e, miR-520f, miR-519c, miR-1283, miR-520a, miR-526b, miR-519b, miR-525, miR-523, miR-518f, miR-520b, miR-518b, miR-520c, miR-518c, miR-524, miR-517a, miR-519d, miR-521, miR-520d, miR-517b, miR-520g, miR-516b, miR-518e, miR-518a, miR-518d, miR-516b, miR-517c, miR-520h, miR-521, miR-522, miR-516a, miR-1283

**miR-379 cluster: **miR-379, miR-411, miR-299, miR-380, miR-1197, miR-323, miR-758, miR-329, miR-494, miR-543, miR-495, miR-376c, miR-376a, miR-654, miR-376b, miR-376a, miR-300, miR-1185, miR-381, miR-487b, miR-539, miR-889, miR-544, miR-655, miR-487a, miR-382, miR-134, miR-668, miR-485, miR-323b, miR-154, miR-496, miR-377, miR-541, miR-409-3p, miR-412, miR-369, miR-410, miR-656

**miR-17 cluster: **miR-17, miR-18a, miR-19a, miR-20a, miR-19b, miR-92a

**miR-424 cluster: **miR-424, miR-503, miR-542, miR-450a, miR-450b, miR-450b

**miR-99b cluster: **miR-99b, let-7e, miR-125a

**miR-106b cluster: **miR-106b, miR-93, miR-25

**let-7a cluster: **let-7a, let-7b

**miR-15b cluster: **miR-15b, miR-16

### In vivo miRNA expression

**Figure 2:**
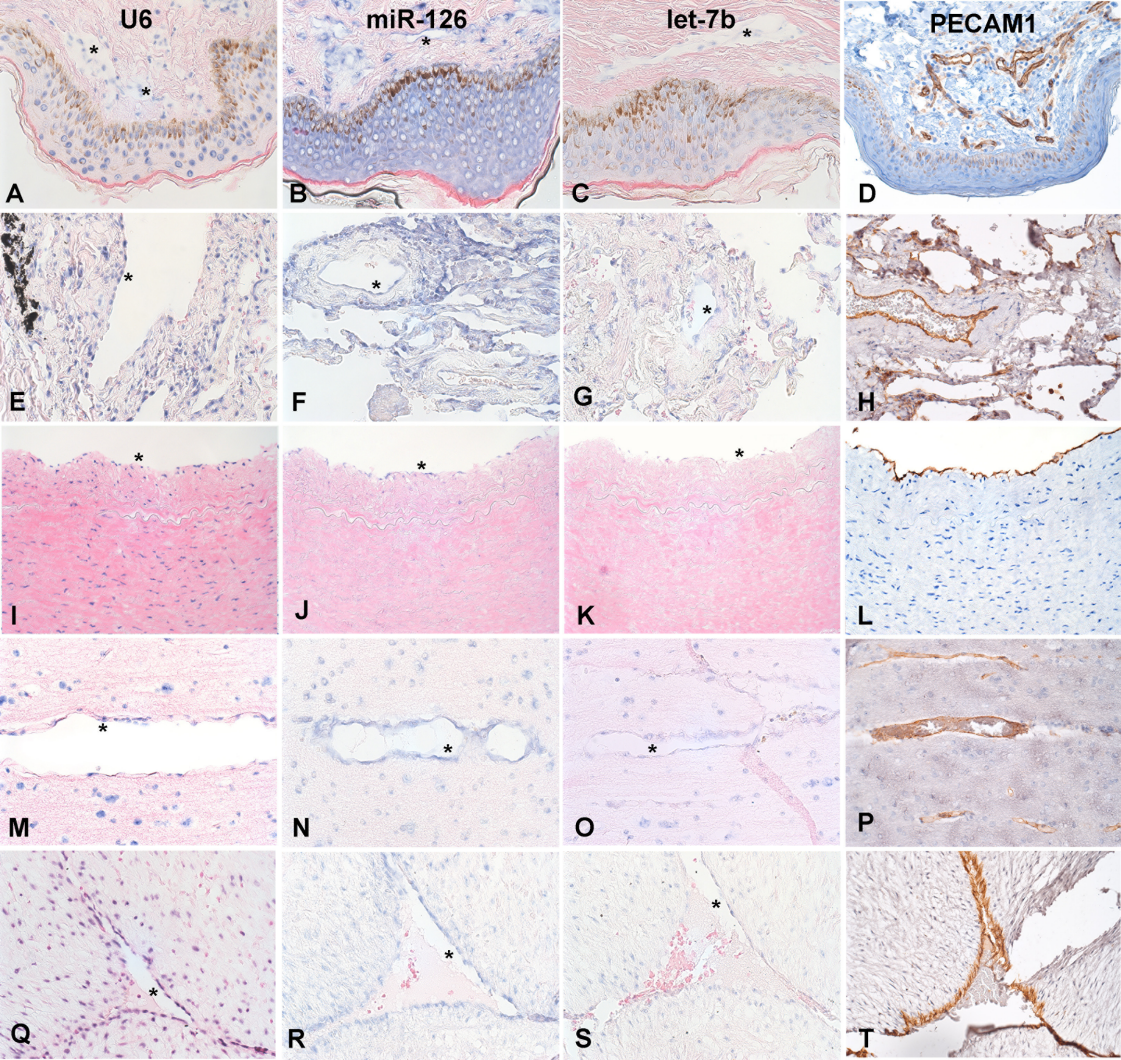
**LNA-ISH of miRNAs miR-126, let-7b and U6 snRNA**. PECAM1 (CD31) staining of endothelial cells. (A-D) Human skin including epidermis, (E-H) human lung, (I-L) coronary artery, (M-P) human brain and (Q-T) human umbilical vein. * represents the location of ECs. For LNA-ISH, positive staining is blue. For PECAM1 IHC, positive staining is brown. Original magnification 160× (A-H, M-T) or 100× (I-L).

We next determined if the miRNAs identified in our expression array could also be seen *in vivo*. We performed LNA-ISH staining of miR-126, let-7b and the control U6 snRNA. Endothelial cells were identified with PECAM1 (CD31) immunohistochemical staining. We identified strong endothelial staining across diverse vascular beds for miR-126. Let-7b was weaker and more variable in the ECs (Figure 2). Of note, miR-126 staining was noticeably stronger in microvessels within a pulmonary lymph node than in the adjacent lymphocytes consistent with the expression data (Additional file 3, Figure S2).

### miRNA predictions based on Sylamer analysis

The mRNA expression patterns of five EC primary cultures, grown similar to our own ECs, have been previously described and are available at Gene Expression Omnibus (GEO - GSE21212) [16]. We used this mRNA data set to determine if we could identify a signature of miRNAs acting on mRNA expression variability among ECs. We used the program Sylamer to identify the representation of 3'UTR miRNA binding sites in genes over or under expressed in a given cell type [18]. Sylamer is a bioinformatics program that catalogs putative miRNA binding sites in the 3'UTR regions of genes and determines if that pattern deviates from neutral expectations in rank-ordered lists of genes.

**Figure 3:**
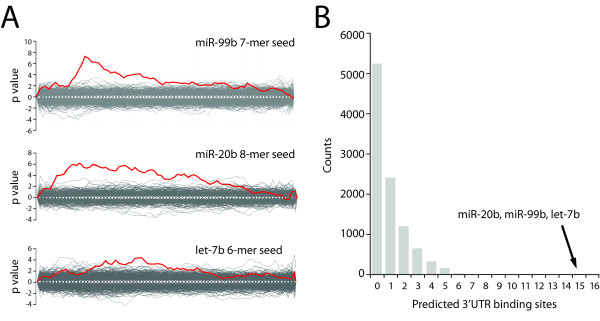
**Sylamer analysis**. (A & B) Representative Sylamer enrichment landscape plots for 3' UTR binding sites for miRNAs. (A) Significant peak for miR-99b 7-mer seed sequence in a comparison of HCECs and HAECs. Significant peak for miR-20b 8-mer seed in a comparison between HCECs and HUVECs. Significant peak for let-7b 6-mer seed in a comparison between HUVEC and HCECs. (B) Resampling analysis histogram of Sylamer 3' UTR binding site data. For all EC comparisons, 162 significant miRNA binding sites were identified. Of these 162 sites, 15 were predicted 3' UTR binding sites for miRNAs miR-20b, miR-99b, and let-7b. A histogram of 10, 000 samplings of 3 random miRNA binding sites was generated to determine the likelihood of having a certain number of predicted 3' UTR binding sites. Having miRNAs miR-20b, miR-99b and let-7b represent 15 binding sites was significantly greater than chance (p < 0.0001).

We began by cataloging all miRNAs that were identified to have a significant enrichment of 3' UTR binding sites in genes variably expressed across any EC comparison (at p < 10^-4^). We performed 11 analyses that compared ECs of different origins (HUVEC vs HAEC, HAEC vs HCEC, etc) for 6, 7, and 8mer bp length miRNA binding sites. We identified 172 instances in which any miRNA 3'UTR binding site was enriched (p ≤10^-4^) across these comparisons. These 172 enrichments were from 107 different miRNAs. Interestingly, the SAM significant miRNAs miR-99b, miR-20b and let-7b were identified 15 times in this group (Figure 3). We determined how likely it was to identify the abundance of these 3 miRNAs by chance by performing a resampling analysis with 10, 000 permutations to determine the likelihood that any combination of 3 miRNAs would be identified 15 times (Figure 3B). No other collection of 3 miRNAs were identified 15 times in the data set, indicating highly significant (p < 0.0001) selection for binding sites for miRNAs miR-99b, miR-20b and let-7b.

We then determined if this signal was biologically relevant. For each comparison of two EC cultures (ex HUVEC vs HAEC) we identified the cell line in which these 3 miRNAs had higher expression. High miRNA expression is associated with lower mRNA expression. In 13 of our 15 comparisons (87%), the culture with higher expressed miRNA had lower expression of genes predicted to have that miRNA binding site.

Sylamer can also be used to identify the genes to which miRNAs bind. We identified 51, 98 and 571 genes under the peaks for miR-99b, miR-20b and let-7b respectively (Figure 3A). The number of genes is a feature of both the sequence motif and the peak width. Known endothelial cell expressed putative targets of miR-99b found under the peak include mechanistic target of rapamycin (*MTOR*), sonic hedgehog (*SHH*), mannin-binding lectin serine peptidase 1 (*MASP1*), and forkhead box O3 (*FOXO3*). Putative targets of miR-20b include polycystic kidney disease 2 (*PKD2*), matrix metallopeptidase 3 (*MMP3*), syndecan 2 (*SDC2*) and fibroblast growth factor receptor substrate 2 (*FRS2*). For let-7b, potential targets include prostaglandin E receptor 3 (*PTGER3*), toll-like receptor 4 (*TLR4*), kruppel-like factor 2 (*KLF2*), and beta 3 integrin (*ITGB3*).

### MiRNA diversity across endothelial, epithelial and hematologic cells

**Figure 4:**
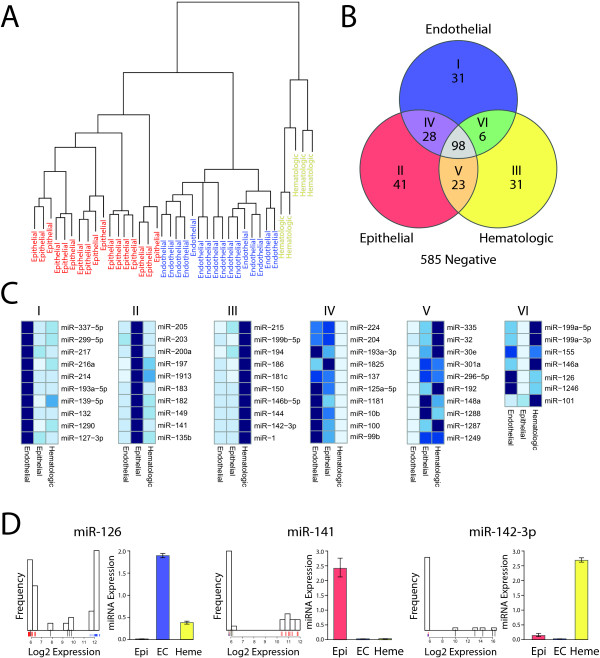
**Endothelial, epithelial and hematologic cell comparisons.** (A) Cluster analysis of endothelial, epithelial and hematologic cells from the same miRNA expression platform based on LIMMA analysis. (B) Venn diagram indicating the complete unique and shared expression patterns of miRNAs between endothelial, epithelial and heamtologic cell types. (C) Representative heat maps from 6 areas of the Venn diagram identifying specific miRNAs in different colored areas. A complete distribution of all miRNA expression is found in the Additional file 4, Table S2. (D) RT-PCR validation of variable expression between cell lines for miRNAs miR-126, miR-141 and miR-142-3p. The left image is a histogram of the frequency of expression from the GEO data set. Epithelial cell lines are red, ECs are blue and hematologic lines are black. The right image is RT-PCR data of the same miRNAs normalized to U6 snRNA.

We then sought to understand how EC miRNA expression related to expression in other cell lineages. We used GEO to identify miRNA datasets generated using Agilent V3 miRNA arrays and methods similar to our own. We obtained data from 17 epithelial and 5 hematologic type cells. Across endothelial, epithelial and hematologic cells, 258 miRNAs were identified that were expressed in at least one cell type. This represents 31% of the human miRNAs present on the Agilent V3 miRNA array. There was marked diversity of miRNA expression resulting in a clear separation of these three different lineages (Figure 4A). Of the expressed miRNAs, 98 miRNAs (38%) were expressed in all cell types (Figure 4B, Additional file 4, Table S2). Thirty-one miRNAs were unique to ECs and an additional 33 were shared with either hematologic or epithelial cells, but not both. Representative heat maps of these different cell comparisons demonstrate these differences (Figure 4C). We also generated a Venn diagram based on LIMMA pairwise comparisons of the 3 cell lineages. This second Venn diagram only investigated miRNAs that were statistically different between at least two cell lineages. Thirty miRNAs, such as miR-126, had expression levels that varied across all 3 cell lineages (Additional file 5 Figure S3, Figure 4D).

**Figure 5:**
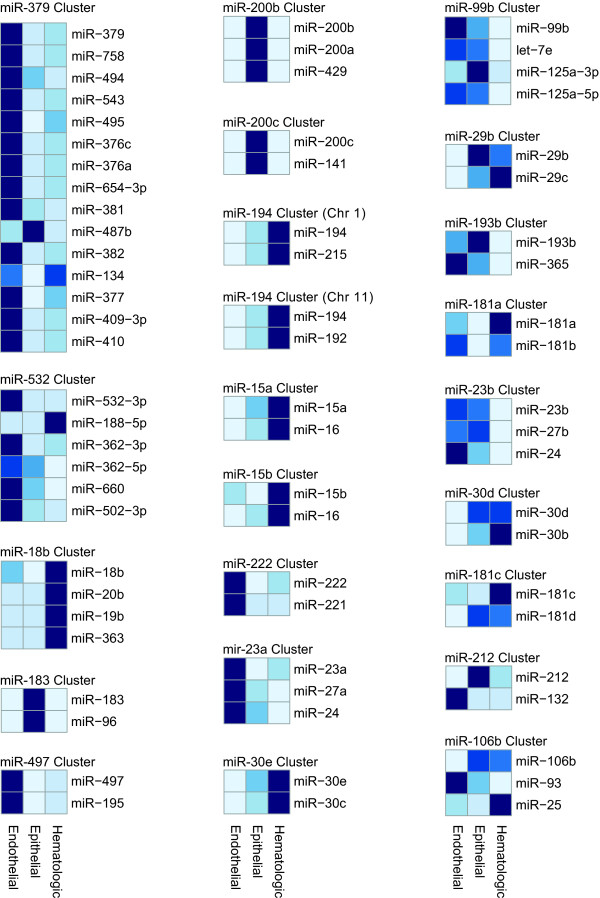
**Heat maps of 23 polycistronic chromosome miRNA clusters**. Each chromosome cluster was selected based on having at least one LIMMA significant pairwise difference. Twenty of 23 chromosome clusters generally display consistent, differential expression between endothelial, epithelial and hematologic cells.

Finally we again wanted to determine if the differences in miRNA expression were explained primarily at the level of transcription. Thus we used the miRNA chromosomal cluster data and identified all clusters that had at least one LIMMA significant difference between cell lineages. Of these 23 clusters, all but 3 (87%) show strong consistent expression across a chromosomal cluster for a given cell type (Figure 5).

### RT-PCR confirmation of 3 cell lineage miRNA array data

We used RT-PCR to validate the array data results across cell types. RT-PCR was performed using a representative EC line (HAEC), epithelial cell line (pancreatic ductal epithelium) and hematologic cells (peripheral blood mononuclear cells). We performed RT-PCR on 6 miRNAs that were found to be dramatically, or subtly variable amongst the three cell lineages. We identified clear validation of the *in silico *results particularly for miRNAs miR-126, miR-141 and miR-142-3p (Figure 4D, Additional file 6, Figure S4).

## Discussion

This is the first study to investigate baseline miRNA diversity in ECs and compare the EC miRNA expression pattern to other major cell types. Using the deep Agilent V3 miRNA array, we found 164 miRNAs expressed in ECs. The vast majority of these miRNAs have similar expression patterns. Given the extent of the similarity, miRNA studies in one EC type (ex HUVEC) should be generally applicable to all EC types. However, we were able to identify three miRNAs, miR-99b, miR-20b and let-7b, which were modestly, but significantly variable among the EC lines by three different types of analysis: LIMMA, SAM and Sylamer analysis. These clustered the EC types into three discrete groups (Figure 1B).

Our data set adds to the understanding of the previously described tissue-wide miRNA studies. For example, miR-126 had relatively high expression in the heart, spleen and thymus and lower expression in the pancreas, colon and fallopian tubes [5]. This correlates with the known relative vascularity of these organs.

Essentially all miRNAs that have previously been described in ECs were in our dataset [19-21]. However, a few were not represented. These miRNAs are generally described as being upregulated by various external stimuli which were not used in this study. This includes miR-663 which is upregulated by shear stress [22] and miR-200c, which is upregulated by oxidative stress [23]. Of the 31 "EC only" miRNAs in our data set, more than half have not previously been identified in ECs and represent new targets for evaluation.

We used our miRNA data to determine if certain types of ECs clustered together. MicroRNA-based clustering has been shown to be a stronger classifier of cancer cell samples than mRNA clustering [24]. We predicted *a priori *that ECs would cluster into macrovascular and microvascular groups. However, the clustering pattern was more complicated. HPAECs and HCECs clustered together, similar to the previously reported mRNA data [16]. HAECs did not cluster with the HPAECs or HCECs, but instead clustered with the microvascular cell cultures HDMVECs and HPMVECs. This was interesting as our HCECs and HAECs were taken from the same individual. We interpret this as evidence that inherited variation is less important to miRNA expression levels than acquired changes based on the vascular location of the ECs. We found that HUVEC and HBMVECs clustered tightly, which was unexpected. We entertained cell passage, the age of the cell donor, and cell confluence as causes of clustering [25-27]. Each was ruled out. The HDMVECs and HUVECs were of fetal origin, the HAECs and HCECs were taken from a young child and the donor's age of the other commercially obtained cell cultures is unknown. All cells were used within passages 3-6. Finally, although all ECs were grown under the exact same conditions, some EC cultures grew faster than others affecting confluency at harvesting (~85-95%). Although some miRNAs are upregulated at higher confluence, miRNAs miR-20b, miR-99b and let-7b have not been shown to be modulated by this effect, and furthermore, there was no consistent pattern of a single EC line, overexpressing a variety of miRNAs [27].

We provided two sets of data that support the transcriptional polycistronic regulation of miRNAs [5]. First, among the ECs, the chromosomal clusters for miRNAs, let-7b, miR-20b and miR-99b all had relative expression patterns that were consistent and supported by RT-PCR of primary transcripts for two clusters (Figure 1C, Additional file 2, Figure S1). Second, the polycistronic miRNAs that varied between endothelial, epithelial and hematologic cells, were almost exclusively (87%) similar in expression patterns for adjacent miRNAs (Figure 5).

There is only limited information on the role of miRNAs let-7b, miR-20b and miR-99b in ECs, although this information is intriguing. MiR-20b is known to regulate estrogen receptor α (*ERα*), nuclear receptor coactivator 3 (*NCOA3/AIB1*) and hypoxia inducible factor 1α (*HIF1α*) [28,29]. It is also decreased in hypoxia [30]. Let-7b regulates tissue inhibitor of matrix metalloproteinase 1 (*TIMP1*) which has anti-angiogenic properties [31]. It also regulates high mobility group at-hook (*HMGA2*), nuclear receptor subfamily 2, group E, member 1 (*NR2E1/TLX*), cyclin D1 (*CCND1*) and is increased in skeletal muscle of older individuals [32-35]. Higher expression of miR-99b is associated with lymph node metastases in esophageal adenocarcinoma [36]. The miR-99b, let-7e, miR-125 cluster modulates *HMGA2 *and SWI/SNF-related, matrix-associated, actin-dependent regulator of chromatin, subfamily A, member 5 (*SMARCA5*) [37].

Our use of Sylamer was novel in a way that bears discussion. Sylamer has traditionally been used in the setting of a specific miRNA knockdown or overexpression to evaluate a targeted set of genes. Reasoning that global changes in mRNA expression would result in miRNA differences, we tested whether Sylamer could be used in a new manner to predict differentially expressed miRNAs. In our experiment there was no overwhelmingly significant p value for a miRNA signature [38]. Rather, sifting through smaller, more frequent changes, we found numerous minor effects. We used the summation of the data to demonstrate a clear increase in miRNA binding sites for let-7b, miR-20b and miR-99b in genes that were variably expressed across EC types in this additional data set. This information, along with the RT-PCR data, help confirm the uniqueness of these 3 miRNAs. Also, most of the peaks we identified for miRNAs miR-99b, miR-20b and let-7b were not at the extreme edge of the graph (Figure 3). This signifies that the most extremely altered genes, are not regulated directly by these miRNAs. Rather they may be downstream of pathways regulated by miRNAs or other regulatory activities in the cell.

We have for the first time established the miRNA expression pattern of ECs relative to epithelial and hematologic cells. Not surprisingly, there were many differences between these cell types. This data will help identify those miRNAs whose activities may regulate functions more intrinsic to one or the other types of cells. It may also assist in determining the origin of miRNAs found in serum [39].

There were a number of limitations to our study. In our comparison of ECs to other cell types, we were limited by the available matching data (same array, same processing software) in GEO. Despite careful sample preparation, processing, and analysis, it is possible that some of the microarray results were affected by technical artifacts introduced by the sample selection, microarray platform, or the hybridization procedure. We addressed this possibility by using two complementary technologies (RT-PCR and Sylamer) to provide further evidence supporting our findings; however, independent replication of the results reported here using different samples and/or a different microarray platform would certainly provide further evidence. The limited amount of publicly available Agilent V3 miRNA microarray data precluded us from generating Gene Expression Barcodes, a more robust method to assess absolute expression [26]. Also, because we only had 3 cell types to compare, it is likely some of the "cell specific" miRNAs can be found in other cell types not contained in our analysis. In our Sylamer data, because multiple miRNAs can bind to the same consensus sequence, the data we present for miR-20b, miR-99b and let-7b could be data for other related miRNAs such as miR-17, miR-100 or another let-7 miRNA.

The expression and relative levels of the EC miRNAs are likely influenced by the choice of matrix material (gelatin), culture media, and lack of other external factors (shear stress, etc) in our experimental design. In an *in vivo *setting, with different extracellular matrices, paracrine signals from adjacent cells, shear stress and other factors, the miRNA expression would be expected to vary from this controlled environment. Thus the generalizability of our findings awaits further study.

## Conclusions

Our study fills a critical need in the developing EC miRNA story. We have, for the first time, fully characterized and catalogued baseline EC miRNA expression from multiple EC locations. We show high similarity between ECs and great diversity between endothelial, epithelial and hematologic cells. We identify three miRNAs (miR-20b, miR-99b and let-7b) that may confer some of the unique phenotypic diversity to ECs and are worthy of further evaluation.

## Methods

### Human primary endothelial cell sources

HAEC and HCEC were harvested from a human aorta and coronary artery taken during cardiac transplantation of a 7-year-old girl. Cells were purified by CD31 magnetic bead separation (Dynal, Carlsbad, CA) and confirmed for EC phenotype by DiI-Ac-LDL staining and CD31 flow cytometry (Additional file 7, Figure S5). HDMVEC (neonatal) were purchased from Cascade Biologics, Invitrogen cell culture (Invitrogen, Carlsbad, CA). HBMVECs, HUVECs, HPAECs and HPMVECs were purchased from ScienCell Research Laboratories (ScienCell, Carlsbad, CA).

### Human primary endothelial cells culture

Human primary endothelial cells were grown on a 2% gelatin matrix with Endothelial Cell Medium (ECM, ScienCell, Carlsbad, CA) supplemented with ECGS (ScienCell, Carlsbad, CA) and 5% FBS (ScienCell, Carlsbad, CA). Cells were grown to 75% confluence, at which time, the media was changed and cells were harvested for RNA 24 hours later, when confluence was ~95%. All cells were between passages 3-6 for these experiments.

### Agilent V3 miRNA array

Total RNA was isolated by miRNeasy kit (QIAGEN, Valencia, CA) according to the manufacturer's instructions. RNA quality was assessed using a Bioanalyser (Agilent, Santa Clara CA). All samples achieved an RNA integrity number (RIN) score greater than 9.5 [40]. RNA samples were then run in duplicate on an Agilent V3 miRNA array according to the manufacturer's instructions in the JHMI Microarray Core Facility. Raw Agilent V3 miRNA data were preprocessed using a modified version of Robust Multi-array Analysis (RMA)[41] without background correction, implemented in the AgiMicroRna R package [42]. This preprocessing method has been shown to have better precision than the preprocessing method recommended by Agilent [43]. The array data has been submitted to GEO (GSE30512).

### miRNA RT-PCR

Total RNA was isolated from human primary endothelial cells, epithelial HPNE cells, and hematologic cells using TRIzol reagents (Invitrogen, Carlsbad, CA) following manufacturer's instructions. RT-PCR was performed with TaqMan^® ^microRNA assays (Applied Biosystems) following the manufacturer's protocol. The thermal cycler's program for reverse transcription was 16°C for 30 minutes, 42°C for 30 minutes and 85°C for 5 minutes followed by 4°C hold. The amplification protocol was 95°C for 10 minutes, 95°C for 15 seconds and anneal/extend at 60°C for 60 seconds, total cycle number is 40. Expression levels were normalized to U6 snRNA by ΔΔCt methods.

### qPCR

For measuring expression of the miR-99b and let-7a primary transcripts, cDNAs were created from total RNA using the QuantiTect reverse transcript kit (Qiagen) following the manufacturers protocol. qPCR was performed using the SYBR Green PCR master mix (Applied Biosystems). Transcript abundance was normalized to β-actin expression. Primer sequences for miR-99b cluster (FW: GCCTGTCTCCTCTGCTTCAC; RV: AGGCCTCCTCACACTCTTCA) and let-7a cluster (FW: CCTGAGCAGGAAGTGAGAGG; RV: CCTCAGTTTCCCCAGGTACA) were designed to amplify a region found within an exon in the annotated Refseq gene proximal to the miRNAs.

### Database mining

Gene Expression Omnibus (GEO, http://www.ncbi.nlm.nih.gov/geo/) was searched for miRNA array datasets that were performed on the Agilent V3 miRNA chip set and analyzed with Agilent software. Sixty-one samples from 4 experiments (GSE22380, GSE25435, GSE23815, GSE24222) were identified of which 22 were "control" or "untreated samples." These 22 samples were from peripheral blood mononuclear cells (PBMCs), CD34+ bone marrow cells, bladder epithelial cells, LNCap cells, and the bladder cancer cell line EJ. All raw data sets were preprocessed using the same method [42].

MiRNA clusters were identified by performing a search at miRBase http://www.mirbase.org for clusters with an inter-miRNA distance of 10, 000 kb. Sixty-six clusters, relevant to the miRNAs present on the Agilent V3 chip, were identified.

EC mRNA dataset GSE21212 was obtained from GEO and normalized using the RMA method in R.

### Venn diagram construction

Two types of Venn diagrams were generated to demonstrate diversity across the different cell types. One Venn diagram was generated by hand curating the normalized expression data of each sample and validating this with deep RNA sequencing reports [44,45] and northern blot images culled from the literature. A second type was generated by identifying miRNAs that were differentially expressed between cell types based on a moderated t-test [46].

### Sylamer analysis

Sylamer was used to analyze miRNA binding site differences in 3'UTR regions of human genes [18]. The analysis was performed using EC mRNA data from the GSE21212 dataset [16]. Average expression levels of 44, 792 gene probes from each cell type (ex. HUVEC, HAEC) were compared between samples and rank ordered by relative expression differences. All EC types were paired (HAEC, HCEC, HDMVEC, HPAEC, HUVEC) in 11 comparisons. The rank ordered probe sets were uploaded into SylArray and analysis was performed against Affymetrix HG_U133_Plus_2 identifiers to maximize the number of genes queried. Statistical significance was assessed using hypergeometric test statistics and a p-value cutoff of ≤10^-4 ^to identify miRNAs whose 3'UTR binding signatures were elevated in any comparison. After all significant miRNAs were identified across 11 comparisons, a resampling analysis was performed. Here, the total number of significant enrichments for the miRNAs miR-20b, miR-99b and let-7b were compared to all other significant enrichments of the other 527 miRNA 3'UTR binding sites. A histogram of the number of significant observations in 10, 000 random assignments of 3 miRNAs was generated to demonstrate the significance of the observed result. The identification of putative 3' UTRs bound by these miRNAs was made by identifying the genes under the peak of each miRNA at the cutoff of ≤10^-4^. The 3' UTR miRNA binding site for miR-20b is GCACTTTG and is shared with miR-17. The 3' UTR miRNA binding site for let-7b is CTACCTCA and is shared with let-7a-i and miR-98. The 3' UTR miRNA binding site for miR-99b is TACGGGTT and is shared with miR-99a and miR-100.

### LNA-ISH and IHC

Locked nucleic acid - In situ hybridization was performed using the protocol of Jǿrgensen et al with modifications as described below [47]. Cases were obtained of lung, coronary artery, placenta, brain, and skin from the surgical pathology files of The Johns Hopkins Hospital. Unstained slides were cut from tissue blocks using RNA precautions. An 8 minute pepsin digest at 37°C (Dako, Denmark) in place of proteinase-K treatment was performed on the slides prior to hybridization. A VWR slide warmer was used instead of a Dako hybridizer for the in situ hybridization step. Levamisole (Dako) was used to inhibit endogenous alkaline phosphatase. Counterstain was performed with 50% eosin (Richard-Allen, Kalamazoo, MI). Double digoxigenin (DIG)-labeled miRCURY LNA detection probes for miR-126, let-7b and U6 snRNA (Exiqon A/S, Denmark) were used.

PECAM1 (CD31) immunohistochemistry was performed using standard IHC protocols. Briefly, 6 micron slides underwent high-temperature antigen retrieval and paraffin removal in Trilogy solution (Cell Marque, Hot Springs, AR) in a pressure cooker. Endogenous peroxidase activity was blocked and slides were incubated with a goat primary PECAM-1 antibody (Santa Cruz Biotechnology, Inc., Santa Cruz, CA) at 1:100 for 30 minutes. Then slides were incubated with a goat HRP polymer conjugate (SuperPicTure, Invitrogen, Camarillo, CA) for 10 minutes, stained with Impact DAB (Vector Labs, Burlingame, CA) for 3 minutes then counterstained with hematoxylin (Richard-Allen Scientific, Kalamazoo, MI).

### Statistical analysis

Differential expression was assessed using two methods: Significance Analysis of Microarrays (SAM) [48] and LIMMA [46]. Pearson's pairwise correlation was used on the normalized values of miRNAs performed in duplicate to determine the relationship between miRNAs within the same chromosome cluster.

## Authors' contributions

MNM performed all statistical analysis of the miRNA data and contributed to the manuscript, OAK performed molecular experiments and contributed to the manuscript, JY performed EC culture, molecular experiments and contributed to the manuscript, KF performed LNA-ISH, ALZ provided intellectual contributions to the design of the experiment and contributed to the manuscript, and MKH conceived the project, performed data analysis, and contributed to the manuscript. All authors have read and approved the final manuscript.

## Pre-publication history

The pre-publication history for this paper can be accessed here:

http://www.biomedcentral.com/1755-8794/4/78/prepub

## Supplementary Material

Additional file 1 **Additional table S1**. LIMMA pairwise differential expression analysis. Click here for file

Additional file 2 **Additional figure S1**. RT-PCR data results for miR-20b, miR-99b and let-7b and pri-miRNA cluster data for let-7a and miR-99 clusters. The miRNA data is normalized to U6 snRNA and the pri-miRNA is normalized to β-actin. Significant differences between each EC comparison are reported in Additional file 1, Table S1. Click here for file

Additional file 3 **Additional figure S2**. miR-126 (A) LNA-ISH staining for miR-126 in a lymph node. The endothelial cell staining (arrows) is much stronger than the background hematopoietic cells. Click here for file

Additional file 4 **Additional table S2**. miRNA expression patterns in endothelial, epithelial and hematologic cells. Click here for file

Additional file 5 **Additional figure S3**. Venn diagram displaying the overlap of differentially expressed miRNAs based on LIMMA pairwise comparisons. Twenty-three miRNAs have expression levels that can discriminate between the three cell types (ex miR-126 as seen in Figure 4D). Thirty-two miRNAs differ between endothelial cells and the other two cell types, thirty-eight miRNAs differ between hematologic cells and the others, and 13 differ between epithelial and the others. Fifty (34+9+7) were differentially expressed in only one comparison, and ninety-nine were not differentially expressed in any of the three comparisons. Click here for file

Additional file 6 **Additional figure S4**. RT-PCR validation of variable expression between cell lines. The left image is a histogram of the frequency of expression from the GEO data set. Epithelial cell lines are red, ECs are blue and hematologic lines are dark yellow. The normalized RT-PCR data of three additional miRNAs (miR-432, miR-210 and miR-186) are seen to the right, sharing the same color scheme as the histograms. The RT-PCR data generally supports the *in silico *results based on normalized GEO dataset values. Click here for file

Additional file 7 **Additional figure S5**. Flow cytometry results for HAECs and HCECs after 4 passages. Flow cytometry for CD31 (PECAM-1) was performed. The blue peak is for CD31 and the red peak is for an isotype control. For HAECs, 99.67% of cells were CD31+ and for HCECs, 97.52% were CD31+. Click here for file
