# Supplementary material for: MicroRNA profiling of diverse endothelial cell types
Source: BMC Med Genomics. 2011 Nov 2;4:78. doi: 10.1186/1755-8794-4-78 (PMC3223144; doi:10.1186/1755-8794-4-78)
Supplement: Additional file 1 — Additional table S1. LIMMA pairwise differential expression analysis. [file 1755-8794-4-78-S1.DOC]

**Additional file 1 – LIMMA pairwise differential expression analysis.**

**Additional Table S1.** Fifty-nine miRNAs were significant in unadjusted LIMMA pairwise differential expression analysis between two EC types. Many miRNAs are significant in more than one comparison. The p value for LIMMA analysis is followed by the p value for the RT-PCR data for let7-b, miR-20b and miR-99b.

| miRNA | Comparison | P value (Array) | P value (RT-PCR) |
| --- | --- | --- | --- |
| let-7a | HDMVEC-HBMVEC | 0.0387 |  |
| let-7a | HPAEC-HDMVEC | 0.0052 |  |
| let-7a | HUVEC-HDMVEC | 0.0263 |  |
| let-7b | HBMVEC-HAEC | 0.0023 | <0.0001 |
| let-7b | HPAEC-HAEC | 0.0396 | <0.0001 |
| let-7b | HUVEC-HAEC | 0.0025 | 0.0005 |
| let-7b | HCEC-HBMVEC | 0.0406 | 0.0006 |
| let-7b | HDMVEC-HBMVEC | 0.0011 | <0.0001 |
| let-7b | HPMVEC-HBMVEC | 0.0034 | 0.0069 |
| let-7b | HDMVEC-HCEC | 0.0440 | N.S. |
| let-7b | HUVEC-HCEC | 0.0448 | 0.0016 |
| let-7b | HPAEC-HDMVEC | 0.0173 | <0.0001 |
| let-7b | HUVEC-HDMVEC | 0.0012 | 0.0003 |
| let-7b | HUVEC-HPMVEC | 0.0037 | N.S. |
| let-7c | HUVEC-HAEC | 0.0491 |  |
| let-7c | HUVEC-HDMVEC | 0.0438 |  |
| let-7c | HUVEC-HPMVEC | 0.0496 |  |
| let-7f | HPAEC-HBMVEC | 0.0312 |  |
| let-7f | HPAEC-HDMVEC | 0.0469 |  |
| let-7g | HPAEC-HBMVEC | 0.0346 |  |
| let-7g | HUVEC-HPAEC | 0.0216 |  |
| let-7i | HPAEC-HBMVEC | 0.0292 |  |
| let-7i | HUVEC-HPAEC | 0.0267 |  |
| miR-15a | HPAEC-HBMVEC | 0.0324 |  |
| miR-15a | HPAEC-HDMVEC | 0.0267 |  |
| miR-15a | HUVEC-HPAEC | 0.0283 |  |
| miR-15b | HPAEC-HBMVEC | 0.0252 |  |
| miR-15b | HDMVEC-HCEC | 0.0123 |  |
| miR-15b | HPAEC-HDMVEC | 0.0048 |  |
| miR-15b | HUVEC-HPAEC | 0.0221 |  |
| miR-16 | HPAEC-HDMVEC | 0.0383 |  |
| miR-17 | HUVEC-HPAEC | 0.0403 |  |
| miR-18b | HPAEC-HBMVEC | 0.0499 |  |
| miR-19a | HPAEC-HBMVEC | 0.0399 |  |
| miR-19a | HUVEC-HPAEC | 0.0418 |  |
| miR-20a | HCEC-HBMVEC | 0.0399 |  |
| miR-20a | HPAEC-HBMVEC | 0.0271 |  |
| miR-20a | HUVEC-HCEC | 0.0312 |  |
| miR-20a | HPAEC-HDMVEC | 0.0444 |  |
| miR-20a | HUVEC-HPAEC | 0.0212 |  |
| miR-20b | HBMVEC-HAEC | 0.0031 | 0.0526 |
| miR-20b | HCEC-HAEC | 0.0367 | 0.0468 |
| miR-20b | HPAEC-HAEC | 0.0101 | 0.0028 |
| miR-20b | HUVEC-HAEC | 0.0010 | 0.0032 |
| miR-20b | HCEC-HBMVEC | 0.0001 | N.S. |
| miR-20b | HDMVEC-HBMVEC | 0.0142 | N.S. |
| miR-20b | HPAEC-HBMVEC | 0.0001 | 0.0175 |
| miR-20b | HPMVEC-HBMVEC | 0.0099 | N.S. |
| miR-20b | HDMVEC-HCEC | 0.0077 | N.S. |
| miR-20b | HPMVEC-HCEC | 0.0109 | N.S. |
| miR-20b | HUVEC-HCEC | 0.0001 | N.S. |
| miR-20b | HPAEC-HDMVEC | 0.0023 | 0.0067 |
| miR-20b | HUVEC-HDMVEC | 0.0042 | N.S. |
| miR-20b | HPMVEC-HPAEC | 0.0032 | N.S. |
| miR-20b | HUVEC-HPAEC | 0.0000 | 0.001 |
| miR-20b | HUVEC-HPMVEC | 0.0030 | 0.049 |
| miR-21* | HDMVEC-HAEC | 0.0190 |  |
| miR-21* | HPAEC-HBMVEC | 0.0324 |  |
| miR-21* | HDMVEC-HCEC | 0.0258 |  |
| miR-21* | HPAEC-HDMVEC | 0.0040 |  |
| miR-21* | HPMVEC-HDMVEC | 0.0076 |  |
| miR-21* | HUVEC-HPAEC | 0.0319 |  |
| miR-22 | HCEC-HBMVEC | 0.0082 |  |
| miR-22 | HPAEC-HBMVEC | 0.0034 |  |
| miR-22 | HDMVEC-HCEC | 0.0056 |  |
| miR-22 | HUVEC-HCEC | 0.0057 |  |
| miR-22 | HPAEC-HDMVEC | 0.0024 |  |
| miR-22 | HUVEC-HPAEC | 0.0024 |  |
| miR-25 | HCEC-HBMVEC | 0.0125 |  |
| miR-25 | HPAEC-HBMVEC | 0.0105 |  |
| miR-25 | HDMVEC-HCEC | 0.0063 |  |
| miR-25 | HUVEC-HCEC | 0.0136 |  |
| miR-25 | HPAEC-HDMVEC | 0.0053 |  |
| miR-25 | HUVEC-HPAEC | 0.0114 |  |
| miR-26a | HDMVEC-HBMVEC | 0.0095 |  |
| miR-26a | HPAEC-HDMVEC | 0.0407 |  |
| miR-26a | HUVEC-HDMVEC | 0.0114 |  |
| miR-29a | HDMVEC-HAEC | 0.0260 |  |
| miR-29a | HPMVEC-HDMVEC | 0.0475 |  |
| miR-29a | HUVEC-HDMVEC | 0.0384 |  |
| miR-30a | HPAEC-HAEC | 0.0489 |  |
| miR-30a | HCEC-HBMVEC | 0.0306 |  |
| miR-30a | HPAEC-HBMVEC | 0.0067 |  |
| miR-30a | HUVEC-HCEC | 0.0399 |  |
| miR-30a | HPAEC-HDMVEC | 0.0197 |  |
| miR-30a | HUVEC-HPAEC | 0.0087 |  |
| miR-30b | HDMVEC-HCEC | 0.0279 |  |
| miR-30b | HPAEC-HDMVEC | 0.0229 |  |
| miR-30d | HDMVEC-HBMVEC | 0.0199 |  |
| miR-30d | HDMVEC-HCEC | 0.0323 |  |
| miR-30d | HUVEC-HDMVEC | 0.0155 |  |
| miR-99b | HCEC-HAEC | 0.0403 | 0.0048 |
| miR-99b | HPAEC-HAEC | 0.0023 | <0.0001 |
| miR-99b | HUVEC-HAEC | 0.0436 | 0.0477 |
| miR-99b | HCEC-HBMVEC | 0.0020 | 0.0196 |
| miR-99b | HPAEC-HBMVEC | 0.0002 | 0.0252 |
| miR-99b | HDMVEC-HCEC | 0.0073 | 0.0302 |
| miR-99b | HPMVEC-HCEC | 0.0398 | 0.0039 |
| miR-99b | HUVEC-HCEC | 0.0011 | N.S. |
| miR-99b | HPAEC-HDMVEC | 0.0005 | N.S. |
| miR-99b | HPMVEC-HPAEC | 0.0023 | 0.0002 |
| miR-99b | HUVEC-HPAEC | 0.0001 | 0.0042 |
| miR-99b | HUVEC-HPMVEC | 0.0442 | 0.0103 |
| miR-100 | HDMVEC-HAEC | 0.0465 |  |
| miR-100 | HDMVEC-HCEC | 0.0113 |  |
| miR-100 | HPAEC-HDMVEC | 0.0073 |  |
| miR-106b | HUVEC-HPAEC | 0.0369 |  |
| miR-125a-3p | HPAEC-HAEC | 0.0483 |  |
| miR-125a-3p | HCEC-HBMVEC | 0.0410 |  |
| miR-125a-3p | HPAEC-HBMVEC | 0.0071 |  |
| miR-125a-3p | HUVEC-HPAEC | 0.0145 |  |
| miR-125b | HDMVEC-HCEC | 0.0484 |  |
| miR-135a* | HCEC-HBMVEC | 0.0175 |  |
| miR-135a* | HPAEC-HBMVEC | 0.0126 |  |
| miR-135a* | HUVEC-HCEC | 0.0341 |  |
| miR-135a* | HUVEC-HPAEC | 0.0244 |  |
| miR-139-5p | HPAEC-HDMVEC | 0.0486 |  |
| miR-151-3p | HPAEC-HBMVEC | 0.0339 |  |
| miR-151-3p | HPAEC-HDMVEC | 0.0239 |  |
| miR-151-3p | HUVEC-HPAEC | 0.0312 |  |
| miR-154* | HCEC-HBMVEC | 0.0416 |  |
| miR-154* | HDMVEC-HBMVEC | 0.0199 |  |
| miR-154* | HPAEC-HBMVEC | 0.0345 |  |
| miR-154* | HUVEC-HCEC | 0.0383 |  |
| miR-154* | HUVEC-HDMVEC | 0.0183 |  |
| miR-154* | HUVEC-HPAEC | 0.0318 |  |
| miR-155 | HDMVEC-HBMVEC | 0.0293 |  |
| miR-155 | HUVEC-HDMVEC | 0.0366 |  |
| miR-188-5p | HPAEC-HAEC | 0.0398 |  |
| miR-188-5p | HPAEC-HBMVEC | 0.0131 |  |
| miR-188-5p | HPAEC-HDMVEC | 0.0205 |  |
| miR-188-5p | HUVEC-HPAEC | 0.0189 |  |
| miR-193a-5p | HCEC-HBMVEC | 0.0172 |  |
| miR-193a-5p | HDMVEC-HBMVEC | 0.0215 |  |
| miR-193a-5p | HPAEC-HBMVEC | 0.0373 |  |
| miR-193a-5p | HPMVEC-HCEC | 0.0416 |  |
| miR-193a-5p | HUVEC-HCEC | 0.0204 |  |
| miR-193a-5p | HUVEC-HDMVEC | 0.0256 |  |
| miR-193a-5p | HUVEC-HPAEC | 0.0444 |  |
| miR-199a-3p | HCEC-HBMVEC | 0.0254 |  |
| miR-199a-3p | HPAEC-HBMVEC | 0.0320 |  |
| miR-199a-3p | HDMVEC-HCEC | 0.0313 |  |
| miR-199a-3p | HUVEC-HCEC | 0.0223 |  |
| miR-199a-3p | HPAEC-HDMVEC | 0.0394 |  |
| miR-199a-3p | HUVEC-HPAEC | 0.0280 |  |
| miR-221 | HPAEC-HDMVEC | 0.0305 |  |
| miR-221 | HDMVEC-HCEC | 0.0477 |  |
| miR-299-5p | HCEC-HBMVEC | 0.0152 |  |
| miR-299-5p | HPAEC-HBMVEC | 0.0254 |  |
| miR-299-5p | HUVEC-HCEC | 0.0194 |  |
| miR-299-5p | HUVEC-HPAEC | 0.0323 |  |
| miR-320b | HPAEC-HBMVEC | 0.0498 |  |
| miR-320c | HBMVEC-HAEC | 0.0371 |  |
| miR-338-3p | HUVEC-HPMVEC | 0.0274 |  |
| miR-374a | HUVEC-HPAEC | 0.0341 |  |
| miR-376b | HUVEC-HCEC | 0.0470 |  |
| miR-424 | HCEC-HBMVEC | 0.0288 |  |
| miR-424 | HPAEC-HBMVEC | 0.0165 |  |
| miR-424 | HDMVEC-HCEC | 0.0094 |  |
| miR-424 | HUVEC-HCEC | 0.0306 |  |
| miR-424 | HPAEC-HDMVEC | 0.0055 |  |
| miR-424 | HUVEC-HPAEC | 0.0175 |  |
| miR-487b | HCEC-HBMVEC | 0.0454 |  |
| miR-487b | HUVEC-HCEC | 0.0420 |  |
| miR-487b | HUVEC-HPAEC | 0.0490 |  |
| miR-494 | HCEC-HBMVEC | 0.0401 |  |
| miR-494 | HPAEC-HBMVEC | 0.0320 |  |
| miR-494 | HDMVEC-HCEC | 0.0421 |  |
| miR-494 | HUVEC-HCEC | 0.0284 |  |
| miR-494 | HPAEC-HDMVEC | 0.0335 |  |
| miR-494 | HUVEC-HPAEC | 0.0226 |  |
| miR-495 | HDMVEC-HBMVEC | 0.0446 |  |
| miR-495 | HUVEC-HDMVEC | 0.0458 |  |
| miR-503 | HDMVEC-HCEC | 0.0463 |  |
| miR-513a-5p | HCEC-HBMVEC | 0.0468 |  |
| miR-513b | HPAEC-HBMVEC | 0.0323 |  |
| miR-513b | HPAEC-HDMVEC | 0.0474 |  |
| miR-513c | HPAEC-HBMVEC | 0.0309 |  |
| miR-513c | HPAEC-HDMVEC | 0.0442 |  |
| miR-542-3p | HCEC-HBMVEC | 0.0458 |  |
| miR-542-3p | HUVEC-HCEC | 0.0309 |  |
| miR-543 | HDMVEC-HBMVEC | 0.0349 |  |
| miR-543 | HUVEC-HDMVEC | 0.0248 |  |
| miR-574-5p | HPAEC-HBMVEC | 0.0191 |  |
| miR-574-5p | HDMVEC-HCEC | 0.0268 |  |
| miR-574-5p | HPAEC-HDMVEC | 0.0079 |  |
| miR-574-5p | HUVEC-HPAEC | 0.0152 |  |
| miR-582-5p | HPAEC-HDMVEC | 0.0451 |  |
| miR-638 | HPAEC-HBMVEC | 0.0466 |  |
| miR-874 | HCEC-HBMVEC | 0.0310 |  |
| miR-874 | HDMVEC-HBMVEC | 0.0111 |  |
| miR-874 | HPAEC-HBMVEC | 0.0193 |  |
| miR-874 | HUVEC-HCEC | 0.0273 |  |
| miR-874 | HUVEC-HDMVEC | 0.0098 |  |
| miR-874 | HUVEC-HPAEC | 0.0170 |  |
| miR-1181 | HUVEC-HDMVEC | 0.0450 |  |
| miR-1225-5p | HPAEC-HBMVEC | 0.0426 |  |
| miR-1225-5p | HUVEC-HPAEC | 0.0409 |  |
| miR-1826_v15.0 | HPAEC-HBMVEC | 0.0309 |  |
| miR-1826_v15.0 | HUVEC-HPAEC | 0.0365 |  |
