# Supplementary figures and images for: MicroRNA profiling of diverse endothelial cell types
Source: BMC Med Genomics. 2011 Nov 2;4:78. doi: 10.1186/1755-8794-4-78 (PMC3223144; doi:10.1186/1755-8794-4-78)

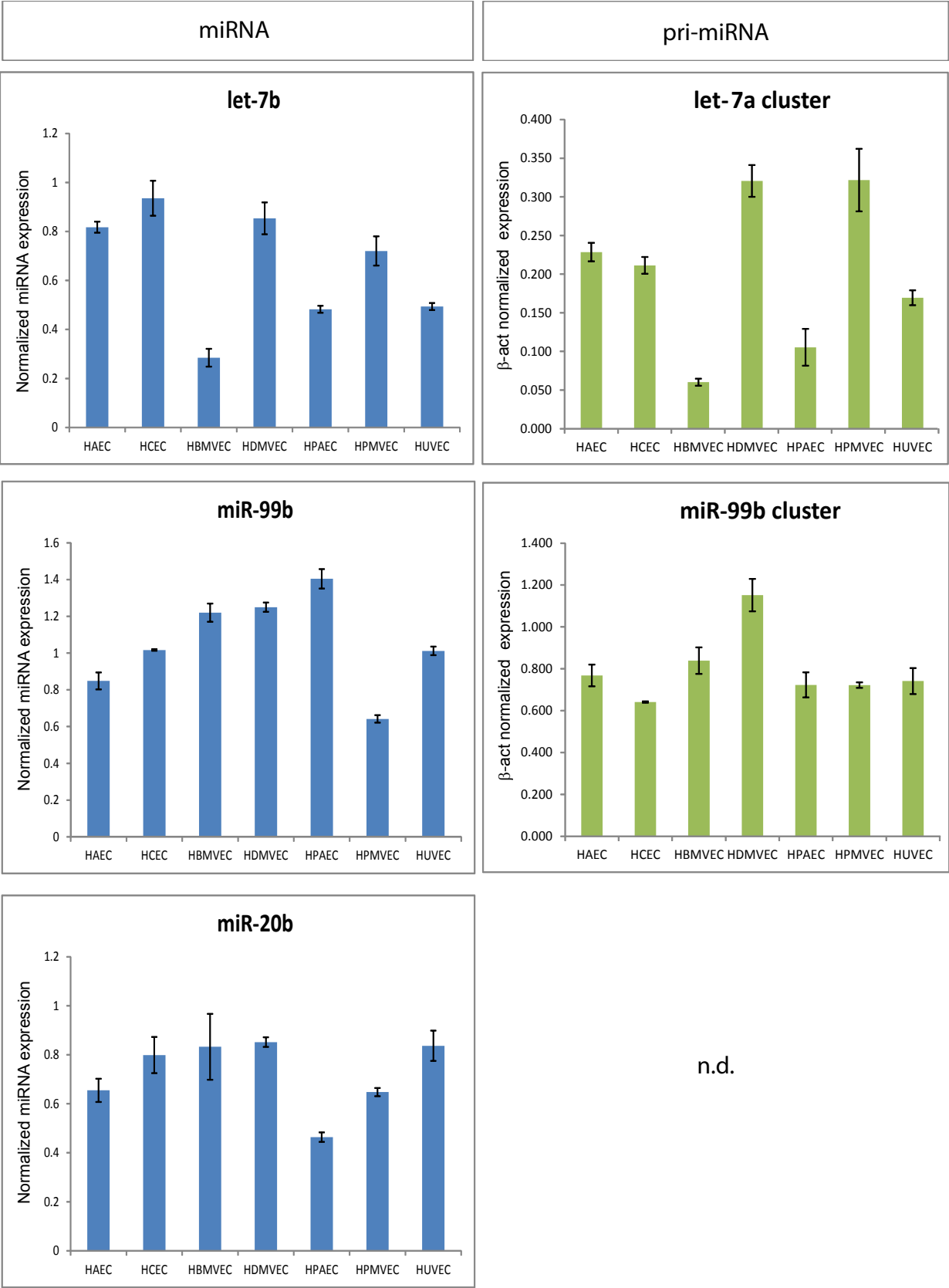

Supplement: Additional file 2 — Additional figure S1. RT-PCR data results for miR-20b, miR-99b and let-7b and pri-miRNA cluster data for let-7a and miR-99 clusters. The miRNA data is normalized to U6 snRNA and the pri-miRNA is normalized to β-actin. Significant differences between each EC comparison are reported in Additional file 1, Table S1. [file 1755-8794-4-78-S2.PDF]

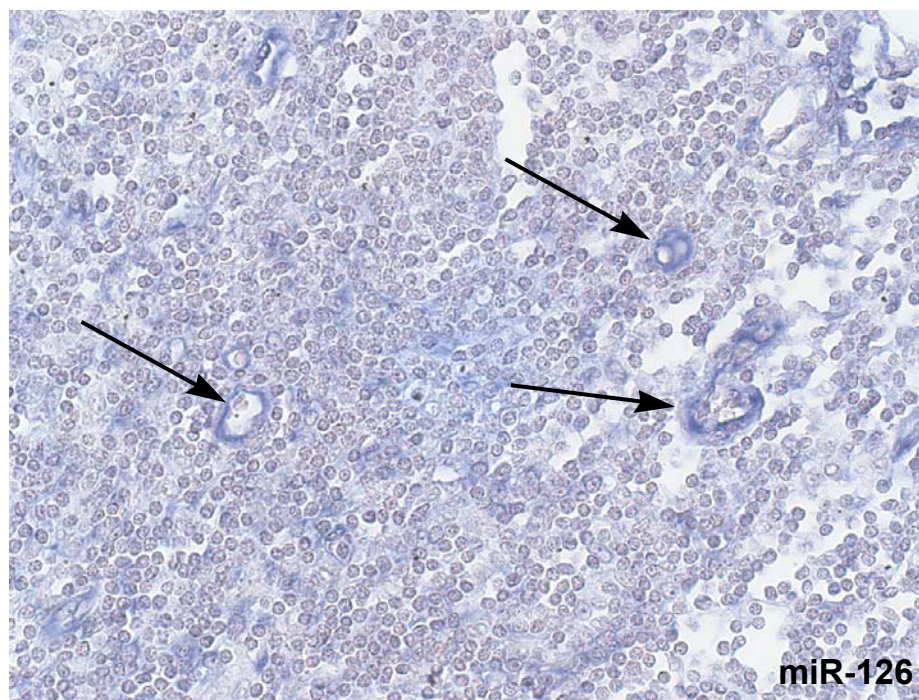

Supplement: Additional file 3 — Additional figure S2. miR-126 (A) LNA-ISH staining for miR-126 in a lymph node. The endothelial cell staining (arrows) is much stronger than the background hematopoietic cells. [file 1755-8794-4-78-S3.PDF]

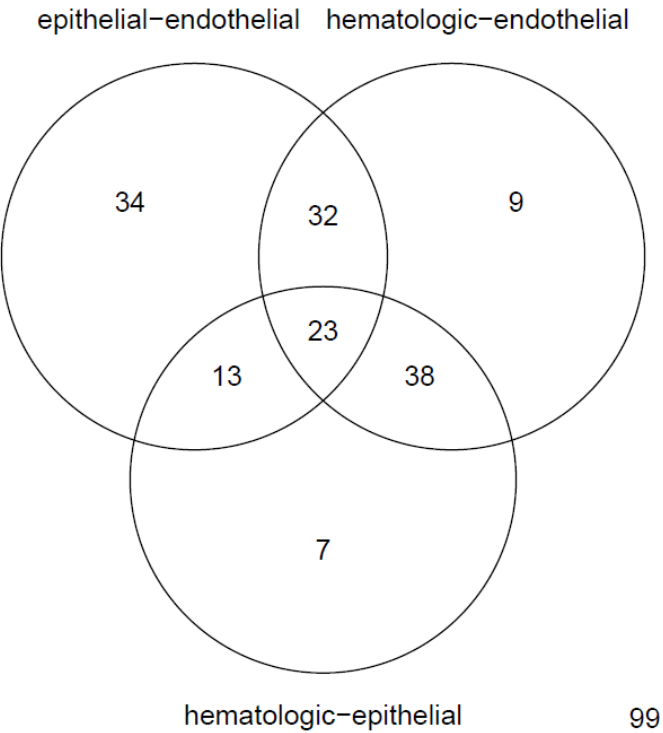

Supplement: Additional file 5 — Additional figure S3. Venn diagram displaying the overlap of differentially expressed miRNAs based on LIMMA pairwise comparisons. Twenty-three miRNAs have expression levels that can discriminate between the three cell types (ex miR-126 as seen in Figure 4D). Thirty-two miRNAs differ between endothelial cells and the other two cell types, thirty-eight miRNAs differ between hematologic cells and the others, and 13 differ between epithelial and the others. Fifty (34+9+7) were differentially expressed in only one comparison, and ninety-nine were not differentially expressed in any of the three comparisons. [file 1755-8794-4-78-S5.PDF]

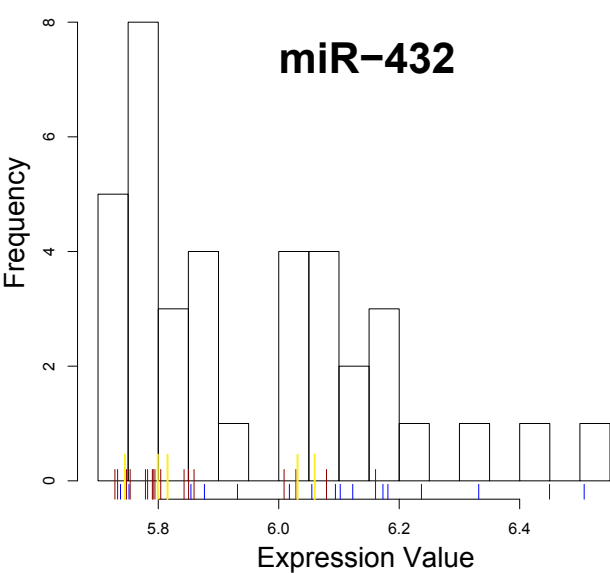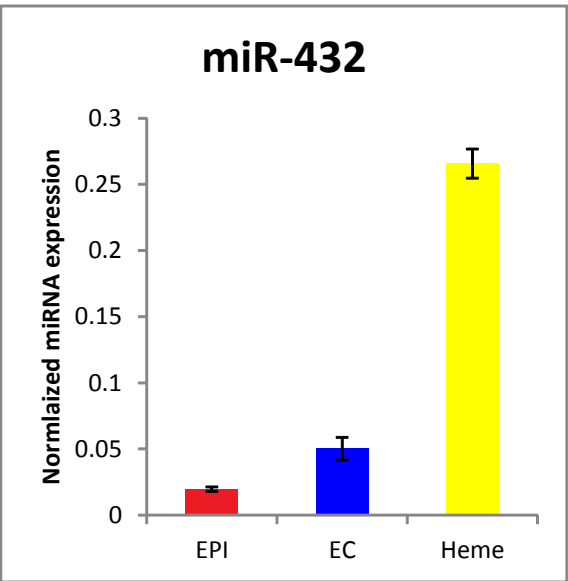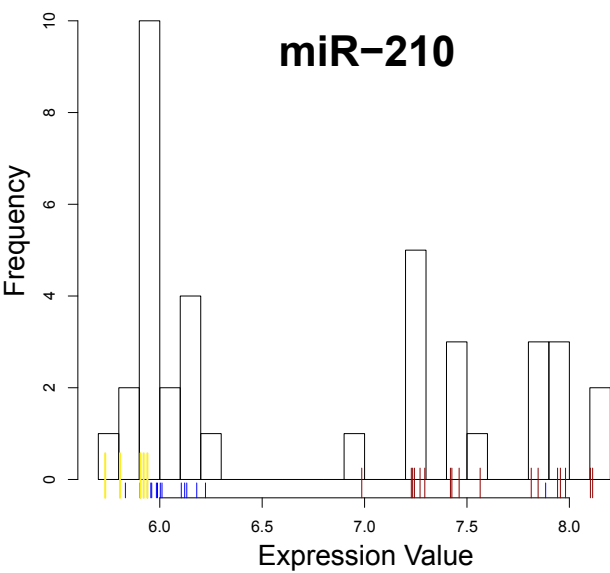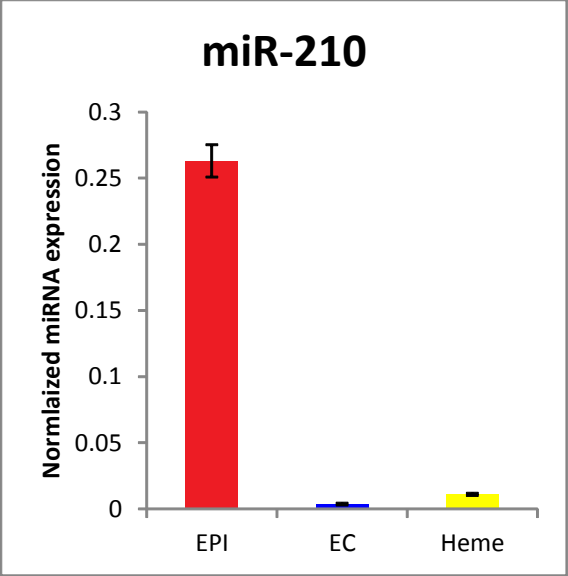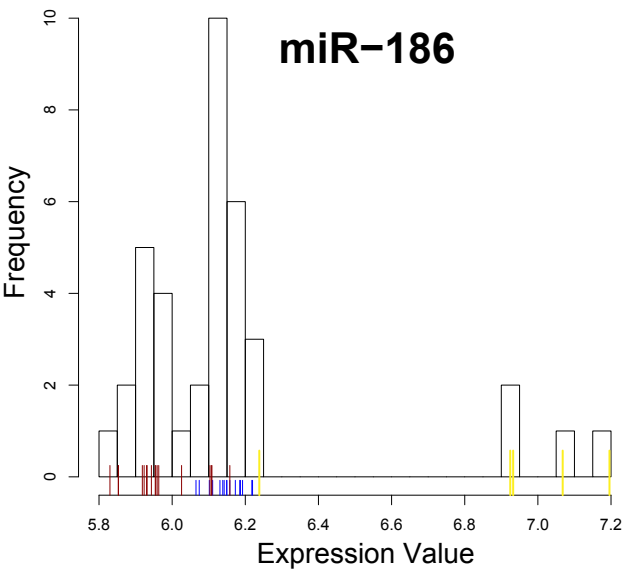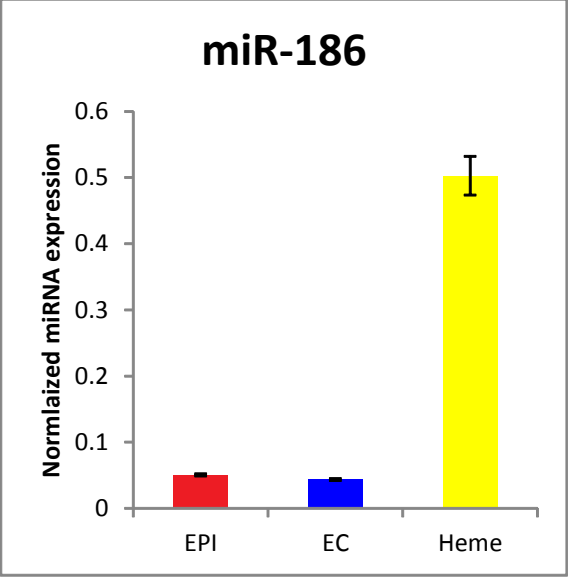

Supplement: Additional file 6 — Additional figure S4. RT-PCR validation of variable expression between cell lines. The left image is a histogram of the frequency of expression from the GEO data set. Epithelial cell lines are red, ECs are blue and hematologic lines are dark yellow. The normalized RT-PCR data of three additional miRNAs (miR-432, miR-210 and miR-186) are seen to the right, sharing the same color scheme as the histograms. The RT-PCR data generally supports the in silico results based on normalized GEO dataset values. [file 1755-8794-4-78-S6.PDF]

Additional File 7  
Title: Additional Figure S5

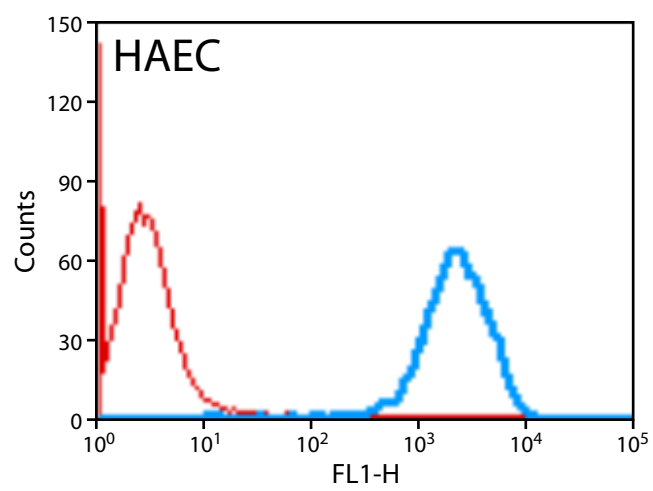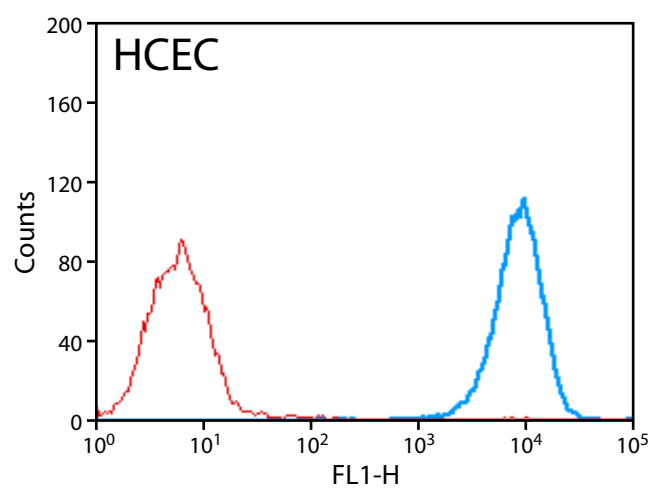

Supplement: Additional file 7 — Additional figure S5. Flow cytometry results for HAECs and HCECs after 4 passages. Flow cytometry for CD31 (PECAM-1) was performed. The blue peak is for CD31 and the red peak is for an isotype control. For HAECs, 99.67% of cells were CD31+ and for HCECs, 97.52% were CD31+. [file 1755-8794-4-78-S7.PDF]
