# Supplementary material for: MicroRNA profiling of diverse endothelial cell types
Source: BMC Med Genomics. 2011 Nov 2;4:78. doi: 10.1186/1755-8794-4-78 (PMC3223144; doi:10.1186/1755-8794-4-78)
Supplement: Additional file 4 — Additional table S2. miRNA expression patterns in endothelial, epithelial and hematologic cells. [file 1755-8794-4-78-S4.DOC]

**Additional file 4**

**Additional Table S2.** The miRNA expression patterns in ECs, epithelial cells and hematologic cells. Data is only presented for miRNAs expressed by at least one cell type.

| miRNA | Shared | EC Only | Epi Only | Heme Only | EC & Epi | EC & Heme | Epi & Heme |
| --- | --- | --- | --- | --- | --- | --- | --- |
| let-7a |  |  |  |  |  |  |  |
| let-7b |  |  |  |  |  |  |  |
| let-7c |  |  |  |  |  |  |  |
| let-7d |  |  |  |  |  |  |  |
| let-7e |  |  |  |  |  |  |  |
| let-7f |  |  |  |  |  |  |  |
| let-7g |  |  |  |  |  |  |  |
| let-7i |  |  |  |  |  |  |  |
| miR-1 |  |  |  |  |  |  |  |
| miR-7-1* |  |  |  |  |  |  |  |
| miR-10a |  |  |  |  |  |  |  |
| mIR-10b |  |  |  |  |  |  |  |
| miR-15a |  |  |  |  |  |  |  |
| miR-15b |  |  |  |  |  |  |  |
| miR-16 |  |  |  |  |  |  |  |
| miR-17 |  |  |  |  |  |  |  |
| miR-17* |  |  |  |  |  |  |  |
| miR-18a |  |  |  |  |  |  |  |
| miR-19a |  |  |  |  |  |  |  |
| miR-19b |  |  |  |  |  |  |  |
| miR-20a |  |  |  |  |  |  |  |
| miR-20a* |  |  |  |  |  |  |  |
| miR-20b |  |  |  |  |  |  |  |
| miR-21 |  |  |  |  |  |  |  |
| miR-21* |  |  |  |  |  |  |  |
| miR-22 |  |  |  |  |  |  |  |
| miR-23a |  |  |  |  |  |  |  |
| miR-23b |  |  |  |  |  |  |  |
| miR-24 |  |  |  |  |  |  |  |
| miR-25 |  |  |  |  |  |  |  |
| miR-26a |  |  |  |  |  |  |  |
| miR-26b |  |  |  |  |  |  |  |
| miR-27a |  |  |  |  |  |  |  |
| miR-27b |  |  |  |  |  |  |  |
| miR-28-5p |  |  |  |  |  |  |  |
| miR-29a |  |  |  |  |  |  |  |
| miR-29b |  |  |  |  |  |  |  |
| miR-29b-1* |  |  |  |  |  |  |  |
| miR-29c |  |  |  |  |  |  |  |
| miR-29c* |  |  |  |  |  |  |  |
| miR-30a |  |  |  |  |  |  |  |
| miR-30a* |  |  |  |  |  |  |  |
| miR-30b |  |  |  |  |  |  |  |
| miR-30c |  |  |  |  |  |  |  |
| miR-30d |  |  |  |  |  |  |  |
| miR-30e |  |  |  |  |  |  |  |
| miR-30e* |  |  |  |  |  |  |  |
| miR-31 |  |  |  |  |  |  |  |
| miR-31* |  |  |  |  |  |  |  |
| miR-32 |  |  |  |  |  |  |  |
| miR-32* |  |  |  |  |  |  |  |
| miR-33a |  |  |  |  |  |  |  |
| miR-33b* |  |  |  |  |  |  |  |
| miR-34a |  |  |  |  |  |  |  |
| miR-34b |  |  |  |  |  |  |  |
| miR-34b* |  |  |  |  |  |  |  |
| miR-92a |  |  |  |  |  |  |  |
| miR-92b |  |  |  |  |  |  |  |
| miR-92b |  |  |  |  |  |  |  |
| miR-93 |  |  |  |  |  |  |  |
| miR-96 |  |  |  |  |  |  |  |
| miR-98 |  |  |  |  |  |  |  |
| miR-99a |  |  |  |  |  |  |  |
| miR-99b |  |  |  |  |  |  |  |
| miR-100 |  |  |  |  |  |  |  |
| miR-101 |  |  |  |  |  |  |  |
| miR-103 |  |  |  |  |  |  |  |
| miR-106b |  |  |  |  |  |  |  |
| miR-107 |  |  |  |  |  |  |  |
| miR-125a-3p |  |  |  |  |  |  |  |
| miR-125a-5p |  |  |  |  |  |  |  |
| miR-125b |  |  |  |  |  |  |  |
| miR-126 |  |  |  |  |  |  |  |
| miR-126* |  |  |  |  |  |  |  |
| miR-127-3p |  |  |  |  |  |  |  |
| miR-128 |  |  |  |  |  |  |  |
| miR-130a |  |  |  |  |  |  |  |
| miR-130b |  |  |  |  |  |  |  |
| miR-132 |  |  |  |  |  |  |  |
| miR-135a* |  |  |  |  |  |  |  |
| miR-135b |  |  |  |  |  |  |  |
| miR-136 |  |  |  |  |  |  |  |
| miR-137 |  |  |  |  |  |  |  |
| miR-139-5p |  |  |  |  |  |  |  |
| miR-140-3p |  |  |  |  |  |  |  |
| miR-140-5p |  |  |  |  |  |  |  |
| miR-141 |  |  |  |  |  |  |  |
| miR-142-3p |  |  |  |  |  |  |  |
| miR-142-5p |  |  |  |  |  |  |  |
| miR-144 |  |  |  |  |  |  |  |
| miR-144* |  |  |  |  |  |  |  |
| miR-146a |  |  |  |  |  |  |  |
| miR-146b-5p |  |  |  |  |  |  |  |
| miR-148a |  |  |  |  |  |  |  |
| miR-148b |  |  |  |  |  |  |  |
| miR-149 |  |  |  |  |  |  |  |
| miR-150 |  |  |  |  |  |  |  |
| miR-151-3p |  |  |  |  |  |  |  |
| miR-151-5p |  |  |  |  |  |  |  |
| miR-155 |  |  |  |  |  |  |  |
| miR-181a |  |  |  |  |  |  |  |
| miR-181b |  |  |  |  |  |  |  |
| miR-181c |  |  |  |  |  |  |  |
| miR-181d |  |  |  |  |  |  |  |
| miR-182 |  |  |  |  |  |  |  |
| miR-183 |  |  |  |  |  |  |  |
| miR-185 |  |  |  |  |  |  |  |
| miR-186 |  |  |  |  |  |  |  |
| miR-191* |  |  |  |  |  |  |  |
| miR-192 |  |  |  |  |  |  |  |
| miR-193a-3p |  |  |  |  |  |  |  |
| miR-193a-5p |  |  |  |  |  |  |  |
| miR-193b |  |  |  |  |  |  |  |
| miR-194 |  |  |  |  |  |  |  |
| miR-195 |  |  |  |  |  |  |  |
| miR-196b |  |  |  |  |  |  |  |
| miR-197 |  |  |  |  |  |  |  |
| miR-199a-3p |  |  |  |  |  |  |  |
| miR-199a-5p |  |  |  |  |  |  |  |
| miR-199b-5p |  |  |  |  |  |  |  |
| miR-200a |  |  |  |  |  |  |  |
| miR-200b |  |  |  |  |  |  |  |
| miR-200c |  |  |  |  |  |  |  |
| miR-203 |  |  |  |  |  |  |  |
| miR-204 |  |  |  |  |  |  |  |
| miR-205 |  |  |  |  |  |  |  |
| miR-210 |  |  |  |  |  |  |  |
| miR-212 |  |  |  |  |  |  |  |
| miR-214 |  |  |  |  |  |  |  |
| miR-215 |  |  |  |  |  |  |  |
| miR-216a |  |  |  |  |  |  |  |
| mIR-217 |  |  |  |  |  |  |  |
| miR-218 |  |  |  |  |  |  |  |
| miR-221 |  |  |  |  |  |  |  |
| miR-222 |  |  |  |  |  |  |  |
| miR-223 |  |  |  |  |  |  |  |
| miR-223* |  |  |  |  |  |  |  |
| miR-224 |  |  |  |  |  |  |  |
| miR-296-5p |  |  |  |  |  |  |  |
| miR-299-5p |  |  |  |  |  |  |  |
| miR-301a |  |  |  |  |  |  |  |
| miR-320a |  |  |  |  |  |  |  |
| miR-320b |  |  |  |  |  |  |  |
| miR-320c |  |  |  |  |  |  |  |
| miR-320d |  |  |  |  |  |  |  |
| miR-324-3p |  |  |  |  |  |  |  |
| miR-324-5p |  |  |  |  |  |  |  |
| miR-328 |  |  |  |  |  |  |  |
| miR-331-3p |  |  |  |  |  |  |  |
| miR-335 |  |  |  |  |  |  |  |
| miR-335* |  |  |  |  |  |  |  |
| miR-337-5p |  |  |  |  |  |  |  |
| miR-338-3p |  |  |  |  |  |  |  |
| miR-340 |  |  |  |  |  |  |  |
| miR-340* |  |  |  |  |  |  |  |
| miR-342-3p |  |  |  |  |  |  |  |
| miR-342-5p |  |  |  |  |  |  |  |
| miR-361-3p |  |  |  |  |  |  |  |
| miR-361-5p |  |  |  |  |  |  |  |
| miR-362-5p |  |  |  |  |  |  |  |
| miR-363 |  |  |  |  |  |  |  |
| miR-365 |  |  |  |  |  |  |  |
| miR-370 |  |  |  |  |  |  |  |
| miR-374a |  |  |  |  |  |  |  |
| miR-374b |  |  |  |  |  |  |  |
| miR-376a |  |  |  |  |  |  |  |
| miR-376c |  |  |  |  |  |  |  |
| miR-377 |  |  |  |  |  |  |  |
| miR-378 |  |  |  |  |  |  |  |
| miR-379 |  |  |  |  |  |  |  |
| miR-381 |  |  |  |  |  |  |  |
| miR-382 |  |  |  |  |  |  |  |
| miR-409-3p |  |  |  |  |  |  |  |
| miR-409-5p |  |  |  |  |  |  |  |
| miR-410 |  |  |  |  |  |  |  |
| miR-421 |  |  |  |  |  |  |  |
| miR-423-5p |  |  |  |  |  |  |  |
| miR-424 |  |  |  |  |  |  |  |
| miR-425 |  |  |  |  |  |  |  |
| miR-425* |  |  |  |  |  |  |  |
| miR-429 |  |  |  |  |  |  |  |
| miR-431 |  |  |  |  |  |  |  |
| miR-432 |  |  |  |  |  |  |  |
| miR-451 |  |  |  |  |  |  |  |
| miR-454 |  |  |  |  |  |  |  |
| miR-455-3p |  |  |  |  |  |  |  |
| miR-483-3p |  |  |  |  |  |  |  |
| miR-484 |  |  |  |  |  |  |  |
| miR-485-3p |  |  |  |  |  |  |  |
| miR-486-5p |  |  |  |  |  |  |  |
| miR-487b |  |  |  |  |  |  |  |
| miR-494 |  |  |  |  |  |  |  |
| miR-495 |  |  |  |  |  |  |  |
| miR-497 |  |  |  |  |  |  |  |
| mIR-500a |  |  |  |  |  |  |  |
| miR-502-3p |  |  |  |  |  |  |  |
| miR-503 |  |  |  |  |  |  |  |
| miR-505 |  |  |  |  |  |  |  |
| miR-513a-5p |  |  |  |  |  |  |  |
| miR-513b |  |  |  |  |  |  |  |
| miR-517a |  |  |  |  |  |  |  |
| miR-517b |  |  |  |  |  |  |  |
| miR-517c |  |  |  |  |  |  |  |
| miR-521 |  |  |  |  |  |  |  |
| miR-522 |  |  |  |  |  |  |  |
| miR-532-3p |  |  |  |  |  |  |  |
| miR-532-5p |  |  |  |  |  |  |  |
| miR-551b |  |  |  |  |  |  |  |
| miR-574-3p |  |  |  |  |  |  |  |
| miR-574-5p |  |  |  |  |  |  |  |
| miR-575 |  |  |  |  |  |  |  |
| miR-582-5p |  |  |  |  |  |  |  |
| miR-584 |  |  |  |  |  |  |  |
| miR-590-5p |  |  |  |  |  |  |  |
| miR-595 |  |  |  |  |  |  |  |
| miR-602 |  |  |  |  |  |  |  |
| miR-625 |  |  |  |  |  |  |  |
| miR-638 |  |  |  |  |  |  |  |
| miR-652 |  |  |  |  |  |  |  |
| miR-654-3p |  |  |  |  |  |  |  |
| miR-660 |  |  |  |  |  |  |  |
| miR-663 |  |  |  |  |  |  |  |
| miR-664 |  |  |  |  |  |  |  |
| miR-671-5p |  |  |  |  |  |  |  |
| miR-758 |  |  |  |  |  |  |  |
| miR-766 |  |  |  |  |  |  |  |
| miR-769-5p |  |  |  |  |  |  |  |
| miR-770-5p |  |  |  |  |  |  |  |
| miR-874 |  |  |  |  |  |  |  |
| miR-885-5p |  |  |  |  |  |  |  |
| miR-887 |  |  |  |  |  |  |  |
| miR-892b |  |  |  |  |  |  |  |
| miR-933 |  |  |  |  |  |  |  |
| miR-939 |  |  |  |  |  |  |  |
| miR-940 |  |  |  |  |  |  |  |
| miR-1181 |  |  |  |  |  |  |  |
| miR-1202 |  |  |  |  |  |  |  |
| miR-1207-5p |  |  |  |  |  |  |  |
| miR-1225-3p |  |  |  |  |  |  |  |
| miR-1225-5p |  |  |  |  |  |  |  |
| miR-1228 |  |  |  |  |  |  |  |
| miR-1234 |  |  |  |  |  |  |  |
| miR-1238 |  |  |  |  |  |  |  |
| miR-1246 |  |  |  |  |  |  |  |
| miR-1249 |  |  |  |  |  |  |  |
| miR-1260 |  |  |  |  |  |  |  |
| miR-1268 |  |  |  |  |  |  |  |
| miR-1275 |  |  |  |  |  |  |  |
| miR-1281 |  |  |  |  |  |  |  |
| miR-1287 |  |  |  |  |  |  |  |
| miR-1288 |  |  |  |  |  |  |  |
| miR-1290 |  |  |  |  |  |  |  |
| miR-1305 |  |  |  |  |  |  |  |
| miR-1539 |  |  |  |  |  |  |  |
| miR-1825 |  |  |  |  |  |  |  |
| miR-1913 |  |  |  |  |  |  |  |
| miR-1914* |  |  |  |  |  |  |  |
| miR-1915 |  |  |  |  |  |  |  |

EC – endothelial cell, Epi – epithelial cell, Heme – hematologic cell, dark box – high expression, dark grey – mixed expression (based on significant difference in LIMMA analysis), light grey – low expression
